# Rubisco substitutions predicted to enhance crop performance through carbon uptake modelling

**DOI:** 10.1093/jxb/erab278

**Published:** 2021-06-11

**Authors:** Wasim A Iqbal, Isabel G Miller, Rebecca L Moore, Iain J Hope, Daniel Cowan-Turner, Maxim V Kapralov

**Affiliations:** 1 School of Natural and Environmental Sciences, Newcastle University, Newcastle Upon Tyne, UK; 2 Lancaster University, UK

**Keywords:** Climate change, crop yield, Earth system models, modelling carbon uptake, photosynthesis, Rubisco

## Abstract

Improving the performance of the CO_2_-fixing enzyme Rubisco is among the targets for increasing crop yields. Here, Earth system model (ESM) representations of canopy C_3_ and C_4_ photosynthesis were combined with species-specific Rubisco parameters to quantify the consequences of bioengineering foreign Rubiscos into C_3_ and C_4_ crops under field conditions. The ‘two big leaf’ (sunlit/shaded) model for canopy photosynthesis was used together with species-specific Rubisco kinetic parameters including maximum rate (*K*_cat_), Michaelis–Menten constant for CO_2_ at ambient atmospheric O_2_ (*K*_c_^21%O2^), specificity for CO_2_ to O_2_ (*S*_c/o_), and associated heat activation (*H*_a_) values. Canopy-scale consequences of replacing native Rubiscos in wheat, maize, and sugar beet with foreign enzymes from 27 species were modelled using data from Ameriflux and Fluxnet databases. Variation among the included Rubisco kinetics differentially affected modelled carbon uptake rates, and Rubiscos from several species of C_4_ grasses showed the greatest potential of >50% carbon uptake improvement in wheat, and >25% improvement in sugar beet and maize. This study also reaffirms the need for data on fully characterized Rubiscos from more species, and for better parameterization of ‘*V*_cmax_’ and temperature response of ‘*J*_max_’ in ESMs.

## Teaser

A state-of-the-art canopy modelling framework was used to predict changes in crop carbon uptake after Rubisco substitutions. We provide a new perspective and improve predictions from previous Rubisco modelling studies at the leaf level.

## Introduction

Climate change has been accelerating since the industrial revolution, with increases in atmospheric CO_2_ leading to higher global temperature (Anwar *et al.*, 2013; Pachauri *et al.*, 2014) The human population has also grown at an unprecedented rate and, with this, human consumption of Earth’s natural resources, resulting in the release of large amounts of carbon into the atmosphere from fossil fuels (Anwar *et al.*, 2013; Pachauri *et al.*, 2014). Without mitigation, this will result in catastrophic consequences. Plants are the foundation of the global food supply and are sensitive to changes in climate as they are major gatekeepers for atmospheric CO_2_. Re-engineering plants to increase carbon uptake through photosynthesis is a target for reducing climate change and increasing global crop yield. Plant CO_2_ uptake is often curbed by a slow turnover rate and the dual affinity of Rubisco for CO_2_ and O_2_ (Ort *et al.*, 2015; Orr and Parry, 2020; Von Caemmerer, 2020). Thus, improving Rubisco performance in crops is among targets for increasing global yields, along with bioengineering photosystems for improving light capture, reductions in photoinhibition, and incorporating carbon-concentrating mechanisms (CCMs) in the C_3_ photosynthesis pathway (McGrath and Long, 2014; Ort *et al.*, 2015; Kromdijk *et al.*, 2016; Evans and Lawson, 2020; Zhu *et al.*, 2020). There is a significant natural variation in Rubisco kinetics between different species (e.g. Hermida-Carrera *et al.*, 2016; Orr *et al.*, 2016; Sharwood *et al.*, 2016; Galmés *et al.*, 2019), and efforts to exploit this to improve crop performance are underway.

To expedite advances in synthetic biology in photosynthesis research, new technologies and community resources across a wide range of platforms need to be developed. These include expanded modelling capacities based on different crop types spanning from leaf up to ecosystem scales (Zhu *et al.*, 2020). Remarkable progress has been made with modelling plant photosynthesis improvements in more realistic settings by upscaling leaf photosynthesis models to the canopy. For example, Zhu *et al.* (2004) showed the consequences of substituting a selection of foreign Rubiscos in a homogenous canopy model, and Wu *et al.* (2019) developed a stand-alone canopy model to assess manipulations in canopy photosynthesis which predicts observed field crop biomass. State of the art Earth system models (ESMs) have been frequently validated against real-world terrestrial carbon fluxes and could also be used to assess the consequences of re-engineering photosynthesis in real-world scenarios for various crop species (Rogers *et al.*, 2017).

In current models, plant photosynthesis is represented by the widely adopted model of Farquhar *et al.* (1980) for C_3_ leaf photosynthesis and that of Collatz *et al.* (1992) for C_4_ photosynthesis. These models are based on Rubisco kinetic parameters frequently measured at 25 °C, including the maximum rate of leaf Rubisco carboxylation (*V*_cmax_), the Michaelis–Menten constant for CO_2_ (*K*_c_) and O_2_ (*K*_o_), and the specificity for CO_2_ to O_2_ (*S*_c/o_). Kinetic parameters are commonly adjusted for changes in temperature using heat activation (*H*_a_) values in an Arrhenius-type equation (Hermida-Carrera *et al.*, 2016). Canopy models first solve the leaf equations of the models of Farquhar *et al.* (1980) and Collatz *et al.* (1992), and then upscale the model inputs to represent an entire plant canopy using the leaf area index (LAI) as a proxy of canopy size, sometimes referred to as the big leaf concept (Rogers *et al.*, 2017; Bonan, 2019). Most canopy models now use the sunlit/shaded canopy modelling approach which is also known as the two big leaf concept. The (dis)advantages have been reviewed (Wu *et al.*, 2016; Rogers *et al.*, 2017).

Although there are canopy modelling platforms available, photosynthesis research is highly interdisciplinary, and progress is constrained by lack of tools on a wide range of platforms. For example, several groups have shown the consequences of substituting different Rubisco species in a tobacco leaf using species-specific Rubisco kinetics and photosynthesis models (see Hermida-Carrera *et al.*, 2016; Orr *et al.*, 2016; Sharwood *et al.*, 2016; Galmés *et al.*, 2019). What remains to be done is assessing the photosynthetic consequences of transplanting Rubisco species in major crops under heterogenous conditions at the canopy level. A few complete Rubisco kinetic datasets are now available, and inclusion of these Rubisco measurements in crop canopy models within platforms used by synthetic biologists would allow rapid identification of ideal Rubisco candidates for a range of scenarios. Here, we developed a single-function canopy model on the R programming platform using a similar methodology to a widely used ESM, the community land model (CLM) 4.5 (Bonan, 2019). The model allowed the consequences of substituting major crop species Rubisco with foreign Rubiscos under real-world field conditions to be evaluated.

## Materials and methods

### Model summary

**Fig. 1.**
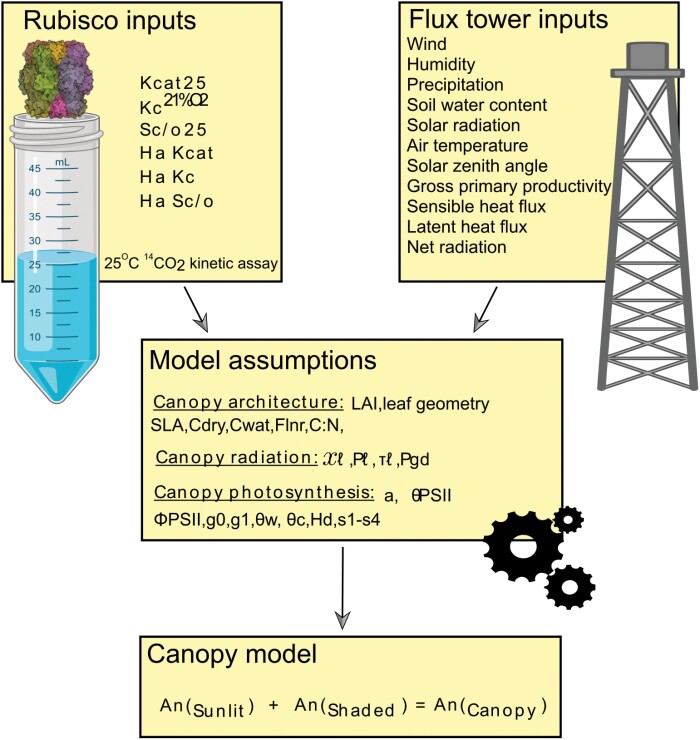
Schematic diagram showing the sunlit/shaded canopy model as a pipeline for testing Rubisco performance in real-world field conditions. Rubisco rates are measured from purified extracts of Rubisco protein in a ^14^CO_2_ fixation assay at 25 °C (see the Materials and methods; Hermida-Carrera *et al.*, 2016). The assay is repeated at multiple temperatures (15, 25, and 35 °C) to produce temperature response curves for each kinetic parameter. Temperature response curves are used to obtain a heat activation value (*H*_a_) for each 25 °C kinetic parameter. Environmental and meteorological data to drive the model simulations are obtained from eddy covariance flux tower measurements located at cropland sites. Model inputs are combined with model assumptions for canopy architecture, the distribution of canopy radiation, and photosynthesis. Sunlit and shaded photosynthesis models are simulated for the duration of the flux tower inputs and then combined to give the final canopy photosynthesis. Abbreviations: *K*_cat25_, maximum turnover rate for CO_2_; *K*_c_^21%O2^25, Michaelis–Menten constant for CO_2_; *S*_c/o_, specificity for CO_2_ compared with O_2_; *H*a *K*_cat_, heat activation for *K*_cat_; H_a_*K*_c_^21%O2^, heat activation for *K*_c_^21%O2^; *H*_a_*S*_c/o_, heat activation for *S*_c/o_; LAI, leaf area index; SLA, specific leaf area; Cdry, heat capacity of dry matter; Cwat, heat capacity of water, Flnr, fraction of leaf nitrogen in Rubisco, C:N, leaf carbon to nitrogen ratio; Xℓ, Ross index; Pℓ; leaf reflectance (visible); τℓ, leaf transmittance (visible); Pgd, soil albedo; *a*, quantum efficiency for C_4_ plants; ΘPSII, smoothing parameter for *J*_max_; фPSII, quantum yield of PSII; *g*0, minimum stomatal conductance; *g*l, maximum stomatal conductance; θ _ w_, field wilting point; θ _c_, saturated soil moisture; *H*_d_, heat deactivation value for C_3_ plants, *s*1–*s*4, heat deactivation values for C_4_ plants; *A*n, net CO_2_ assimilation (see Supplementary Table S1). Rubisco structure shown for a Form-I Rubisco (10.2210/pdb5WSK/pdb). The image of the test tube was obtained from Biorender (Biorender.com).


The sunlit/shaded approach was adopted which is used in many ESMs such as the CLM (Rogers *et al.*, 2017). The sunlit/shaded approach involves calculating the photosynthetic rate of a plant’s sunlit and shaded regions separately and then summing them to give the total photosynthetic rate (Fig. 1). To drive the models of photosynthesis requires canopy temperature, intercellular CO_2_ (*C*_i_), and photosynthetically active radiation (PAR) as inputs. Canopy temperature was obtained from balancing an energy budget equation (Supplementary Protocols) (Bonan *et al.*, 2011; Bonan, 2019). PAR for the shaded and sunlit region was solved analytically using the two-stream approximation radiative transfer model which calculates canopy PAR profile as a function of canopy architecture (i.e. leaf angle orientation and LAI), leaf optical properties, environmental direct beam radiation, and diffuse beam radiation (Supplementary Protocols) (Liou, 2002; Bonan *et al.*, 2011; Bonan, 2019). *C*_i_ was solved numerically using a hybrid approach involving both Newton–Raphson and bisection root-finding methods (Supplementary Protocols) (Sun *et al.*, 2012; Burden, 2016). Leaf parameters including Rubisco kinetics obtained from Flamholz *et al.* (2019), stomatal conductance, and PAR were scaled to sunlit and shaded fractions of the canopy. Rubisco *V*_cmax_ (and the corresponding light-limited rate *J*_max_) was upscaled to the canopy using LAI and partitioned across sunlit/shaded regions of the canopy (Supplementary Protocols). Canopy temperature, *S*_c/o_, and *K*_c_ were assumed to be the same for both sunlit and shaded regions. Supplementary Table S1 shows all the constants and parameters used. The model was packaged as a single user-friendly function (https://github.com/Iqbalwasim01/Sunlit-shaded-canopy-photosynthesis-model.git):

$$\begin{array}{l} \frac{An}{dt}=Two.big.leaf.concept(\ldots ) \end{array}$$ (1)

where the R function ‘Two.big.leaf.concept(…)’ takes all of the required environmental, canopy architecture and Rubisco kinetic inputs for a single time point . The function then returns the canopy net CO_2_ assimilation *A*n/d*t* for that time point. Inputs for multiple time points can be introduced by providing field names from a data frame.

### Description of site data for simulations

The environmental data to parameterize and test simulations, including half hourly meteorology and CO_2_ flux measurements, were taken from Fluxnet (https://fluxnet.org/) and Ameriflux (https://ameriflux.lbl.gov/) databases for maize, wheat, and sugar beet cropland sites. The US-Bo1 site (US-Bo1: latitude 40.0062, longitude –88.2904) was planted with *Zea mays* L. (maize) with a maximum reported LAI of 4.21 during 2005 and 2007 from May to September (Bernacchi *et al.*, 2006). The IT-Ca2 site has rotated between grassland and *Triticum aestivum* L. (winter wheat) (IT-Ca2: latitude 42.37722, longitude 12.02604). Winter wheat had an average maximum LAI of 2.27 (derived from the MODIS satellite LAI product) and was planted in November 2013 and harvested in July 2014 (Sabbatini *et al.*, 2016). The BE-Lon site (BE-Lon: latitude 50.55162, longitude 4.74623) was planted with *Beta vulgaris* L. (sugar beet) with a maximum reported LAI of 4 during April 2004 and harvested in September 2004 (Moureaux *et al.*, 2006). For Rubisco simulations, half-hourly meteorology and CO_2_ flux measurements were averaged to give daily measurements with a solar radiation limit ≥5 W m^–2^ to remove night-time observations. For sensitivity analyses, half-hourly measurements were bin averaged by hour to give an averaged diurnal period for the entire growing season. This serves to reduce random variation from carbon flux measurements and makes changes to input parameters easily visible (Houborg *et al.*, 2013).

Seasonal LAI trends for each site were approximated retrospectively using the growing production day (GDP) method which produced the least error in simulations compared with two other retrospective LAI retrieval methods (see Supplementary Table S2) (Xin *et al.*, 2019). The GDP method uses estimates of gross primary productivity (GPP) at each time point of the site as a proxy of LAI. The reported GPP is converted to LAI using a conversion ratio (m^2^ m^–2^ per µmol CO_2_ d^–1^). The conversion ratio was calculated from the point at which maximum GPP was achieved in the growing season divided by the maximum reported LAI (Xin *et al.*, 2019). The final LAI for each day was then calculated as a weighted mean of the previous daily LAI so that LAI is lagging behind the steady state, since physiologically photosynthesis does not instantaneously lead to biomass accumulation (Xin *et al.*, 2019).

### Model parameterization

#### Canopy temperature

Solar radiation will heat a canopy, and the frequency at which a canopy dissipates the heat will depend on environmental conditions such as wind and the conductivity of the canopy’s material, which is quantitatively known as the heat capacity (see Supplementary Protocols) (Bonan *et al.*, 2011; Bonan, 2019). In the CLM and other ESMs, the amount of energy available for the canopy is the net energy from balancing sensible heat flux, latent heat flux, and net radiation (which can be obtained from flux site measurements) (Bonan *et al.*, 2011; Bonan, 2019). Canopy temperature was assumed to be the same for both sunlit/shaded fractions of the canopy.

#### Photosynthetically active radiation

Solar radiation used by the crops for photosynthesis was modelled using the two-stream approximation radiative transfer model (see Supplementary Protocols) (Liou, 2002; Bonan *et al.*, 2011; Bonan, 2019). The two-stream approximation calculates the downward transmission and reflectance of solar radiation in a canopy using the leaf optical properties, soil albedo, LAI, and the angle of the solar radiation (Liou, 2002; Bonan *et al.*, 2011; Bonan, 2019). The angle of the irradiance was based on assuming the leaf angle of crop species using the Goudriaan (1988) function and the corresponding Ross indices for crops which describe the departure of leaf angles from a spherical distribution (Supplementary Table S1). The overall solar radiation absorbed by the canopy was obtained from the overall radiative balance between the upward and downward canopy solar radiation fluxes in the visible wavelength. The total canopy solar radiation was partitioned between sunlit and shaded (see Supplementary Protocols). Sunlit and shaded solar radiation were converted to sunlit and shaded PAR by multiplying by 4.6 (µmol PAR W m^–2^).

#### Intercellular CO_2_

Photosynthesis models must be coupled to a model which quantifies the amount of atmospheric CO_2_ (*C*_a_) taken up by the plant required for carbon fixation (see Supplementary Protocols). This step is challenging because calculating the amount of CO_2_ that reaches the site of carboxylation—the chloroplast CO_2_ concentration (*C*_c_)—is also dependent on the model output (i.e. carbon fixation) and must consider the plant boundaries of *C*_a_ to *C*_c_; the leaf boundary layer conductance (*g*_b_), stomatal conductance (*g*_s_), and mesophyll conductance (*g*_m_), which are also dependent on carbon fixation. Furthermore, because CO_2_ diffusion depends on a multitude of model parameters which differ between sunlit and shaded fractions, CO_2_ diffusion was solved for both the sunlit and shaded fractions separately. Below is Equation 2 that quantifies the relationship between these plant boundaries, gross CO_2_ assimilation (*A*), *C*_c_, and *C*_a_:


$$\begin{array}{l} C_{c}=C_{a}-\frac{A}{g_{b}}-\frac{A}{g_{s}}-\frac{A}{g_{m}}\;\;\; \end{array}$$ (2)

ESMs simplify Equation 2 by ignoring *g*_m_ as it remains unclear how to parameterize this in ESMs, and instead intercellular CO_2_ (*C*_i_) is considered the final amount of *C*_a_ in the plant (Rogers *et al.*, 2017). However, it is important to note that *g*_m_ and photorespiration refixation differ between species throughout the canopy and may lead to marked changes in photosynthesis (Busch *et al.*, 2013; Von Caemmerer and Evans, 2015, Clarke *et al.*, 2021). *g*_s_ is calculated using stomatal models in ESMs (Franks *et al.*, 2017). The Ball and Berry stomatal model is widely used because of its simplicity (Bonan *et al.*, 2011; Franks *et al.*, 2017). *g*_b_ is calculated using equations from engineering studies which quantify the conductivity of the leaf/canopy surface based on environmental wind and leaf/canopy thickness (see Supplementary Protocols). Finally, to solve the non-linear nature of ‘*A*’, a numerical approach involving the Newton–Raphson and bisection root-finding methods is used (see Supplementary Protocols) (Sun *et al.*, 2012; Burden, 2016). Briefly, the root-finding methods begin with an initial approximate for *C*_i_ which is fed through a series of equations to recalculate *C*_i_, giving a new *C*_i_ guess. If the difference between the initial guess and the new *C*_i_ is below a convergence criterion (i.e. <0.001 µmol m^–2^), the process is stopped and the new *C*_i_ is assumed to be the solution, otherwise the process continues until the convergence criterion is met.

### Rubisco kinetics, distribution in the canopy, and temperature dependency

Rubisco performance is quantified by kinetic parameters including the maximum turnover rate (*K*_cat_) (a component of *V*_cmax_), *K*_o_, *K*_c_, and *S*_c/o_, and a *H*_a_ value for each kinetic parameter needed for temperature adjustments. An initial search for *in vitro* kinetics measured in the same conditions (near pH 8 and 25 °C) were obtained from Flamholz *et al.* (2019). Studies which did not have *H*_a_ values were removed. *K*_c_ adjusted to ambient O_2_ (*K*_c_^21%O2^) was used, which avoided the need for *K*_o_ measurements for modelling (Supplementary Table S1). Two studies reporting 27 distinct Rubisco variants were included in the final simulations (Hermida-Carrera *et al.*, 2016; Sharwood *et al.*, 2016).

Scaling the Rubisco parameters from the leaf to the canopy was done as follows: *S*_c/o_ and *K*_c_^21%O2^ kinetics remained unchanged (see also Supplementary Protocols); and *V*_cmax_ (and its corresponding light-limited rate *J*_max_) at the top of the canopy per leaf area was calculated from *K*_cat_ and nitrogen content (*N*_a_) as described by Houborg *et al.* (2013). *V*_cmax_ (and its corresponding light-limited rate *J*_max_) at the top of the canopy per leaf area was then partitioned across the canopy based on the sunlit and shaded fractions of the LAI, and the distribution of *N*_a_ throughout the canopy was quantified by a nitrogen extinction coefficient (*K*_n_=0.3). *K*_n_ was assumed to be the same for all crops as it is uncertain how to vary *K*_n_ for different growth stages/environments in ESMs (Bonan *et al.*, 2012; Hikosaka *et al.*, 2016). Models of photosynthesis require additional parameters including the light-limited rate, *J*_max_, dark respiration, *R*_d_, and an initial slope of the CO_2_ response curve for C_4_ plants, *K*_p_, which have been found to have a relationship to *V*_cmax_ (see Supplementary Protocols) (Collatz *et al.*, 1992; Sellers *et al.*, 1992; Medlyn *et al.*, 2002). For *J*_max_, it was assumed that it was 1.67 *V*_cmax_, for *R*_d_ it was assumed that it was 0.015 *V*_cmax_ for C_3_ plants and 0.025 *V*_cmax_ for C_4_ plants, and for *K*_p_ it was assumed that it was 20 000 *V*_cmax_ (Collatz *et al.*, 1992; Sellers *et al.*, 1992; Medlyn *et al.*, 2002).

The Arrhenius equation with *H*_a_ values was used to adjust Rubisco kinetics for temperatures other than 25 °C (see Supplementary Protocols). For parameters that co-varied with *V*_cmax_, these were adjusted for temperature according to changes in *V*_cmax_, except *J*_max_. It is well known that the temperature response of *J*_max_ is closely linked to the properties of the host plant including the thylakoid membrane and lipid composition (Von Caemmerer, 2000; Leuning, 2002). Leuning (2002) demonstrated that the temperature response of *J*_max_/*V*_cmax_ shows little variation among species for <30 °C. Therefore, all C_3_ crops in this study used the same *H*_a_ value for *J*_max_ (Supplementary Table S1). Different temperature functions were used to reduce Rubisco performance at extreme temperatures for C_3_ and C_4_ species (Farquhar *et al.*, 1980; Sellers *et al.*, 1992).

#### Canopy photosynthesis model

This model incorporated the model of Farquhar *et al.* (1980) for C_3_ photosynthesis and that of Collatz *et al.* (1992) for C_4_ photosynthesis (see Supplementary Protocols). Briefly, both models assume that photosynthesis is the minimum of three rate-limiting steps:


$$\begin{array}{l} A_{n}=min(A_{c},A_{j},A_{p})R_{d} \end{array}$$ (3)

Canopy net photosynthesis, *A*n, is the minimum of the Rubisco-limited rate *A*c, the light-limited rate *A*j, and the phosphate-limited rate *A*p for C_3_ plants or phosphoenolpyruvate (PEP) carboxylase rate for C_4_ plants minus dark respiration (*R*_d_). In nature, the transitions between the *A*c and *A*j rates do not occur instantaneously, thus quadratic functions by Collatz *et al.* (1992) were used to smooth the transition between the *A*c and *A*j rates with different smoothing parameters for C_3_ and C_4_ species.

### Model validation and sensitivity

Canopy models with crop site native Rubisco species kinetics were compared with the observed daily net ecosystem exchanges (NEEs) (µmol CO_2_ m^–2^ d^–1^) of cropland sites to assess the reproducibility of the models. Model accuracy was summarized using the residual mean squared error (RMSE), mean absolute error (MAE), and coefficient of determination (*R*^2^).

The model that resulted in the least accuracy which would have the greatest potential for improvement was used to explore the sensitivity of the canopy model to key input parameters. Parameters which affect the most important parameter, *V*_cmax_, that are assumed to be static at the leaf level in each model but are highly variable in nature, including specific leaf area (SLA), *N*_a_, and fraction of leaf nitrogen in Rubisco (Flnr), were altered individually while keeping other parameters unchanged (see Supplementary Protocols).

### Substitution of crop Rubiscos

Canopy models with crop site native Rubisco species kinetics were compared with canopy models with foreign Rubisco species kinetics rather than a direct comparison with the NEE observations of the flux sites. This was to ensure any errors arising from modelling were accounted for in the simulations. The performance comparison was summarized as the total net carbon uptake per growing season. Statistical testing was not performed to determine the significance of performance differences between Rubisco species, because all simulations included the same margin of error identified in the model validation stage. Therefore, any differences in the simulations will be because of changes in Rubisco species.

### Simulation process

Daily environmental conditions, canopy architecture (i.e. LAI, leaf orientation, SLA, Flnr), and species-specific Rubisco kinetics (i.e. *K*_cat_, *S*_c/o_, *K*_c_^21%O2^, and associated *H*_a_ values) were inputs into the model (Fig. 1). Canopy temperature, PAR, and *C*_i_ diffusion were simulated using the model inputs. Canopy temperature, PAR, and *C*_i_ diffusion were used to obtain daily net CO_2_ assimilation values. The model process was repeated using each set of species-specific Rubisco kinetics and associated *H*_a_ values for each flux site.

### Phylogenetic analysis of Rubisco species

Protein sequences of the Rubisco large subunit (RbcL) for species included in this study were obtained from gthe NCBI protein database. Alignment of Rubisco species was conducted with the ‘ClustalOmega’ algorithm using the ‘msa’ R package (version 1.22.0) (Bodenhofer *et al.*, 2015). A phylogenetic tree was produced from the multialignment using the ‘ggtree’ R package (version 2.4.1) (Yu, 2020).

## Results

### Phylogeny of Rubisco species

**Fig. 2.**
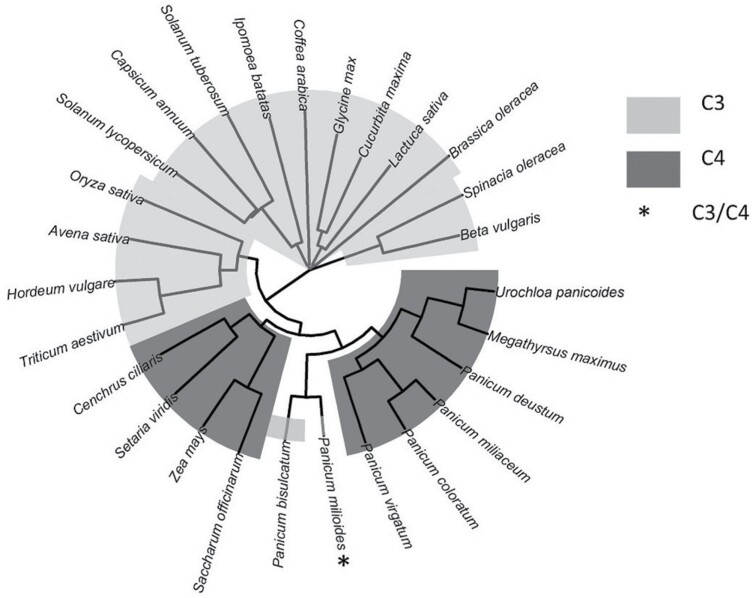
Phylogenetic tree of species included in this study. Of the kinetics of 217 Rubisco species reported in the literature, only 27 Rubisco species had sufficient data for this study (Flamholz *et al.*, 2019). Of 27 Rubisco species, 16 are from C_3_ plants, 10 from C_4_ plants, and one from a C_3_/C_4_ intermediate plant.

Only a fraction of total Rubisco kinetic data found in the literature was included in this study (Fig. 2). The majority of the Rubisco species included were from C_3_ plants.

### Model validation

**Fig. 3.**
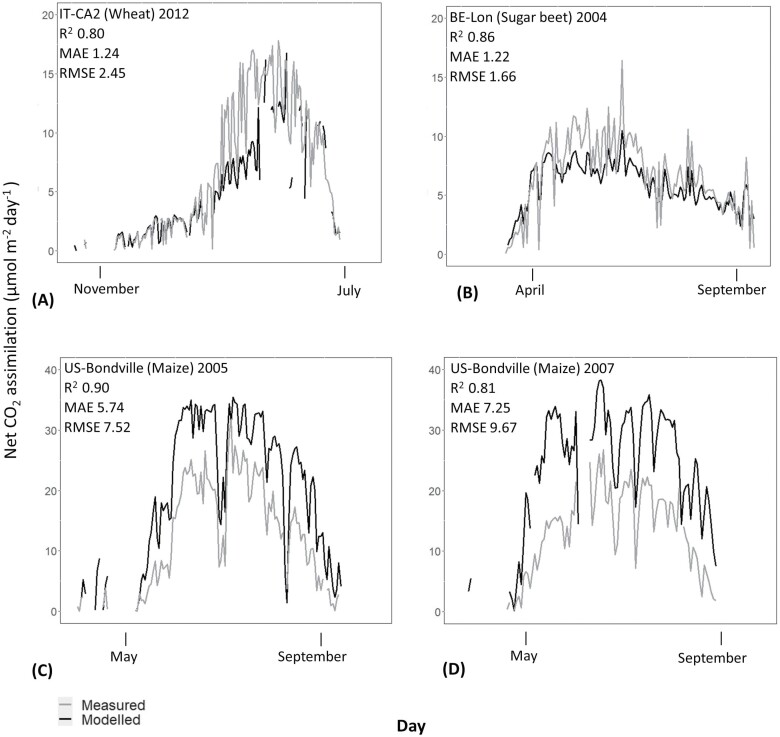
Comparison between modelled (black line) and observed (grey line) daily net CO_2_ assimilation (µmol m^–2^ d^–1^). Measures of error include residual mean squared error (RMSE), mean absolute error (MAE), and coefficient of determination (*R*^2^). Gaps in the modelled growing seasons occurred when environmental or meteorological data were not available.

Simulations with crop site species-specific Rubisco kinetics for wheat, sugar beet, and maize had shown high agreement with observed NEEs (*R*^2^=0.80–0.90) (Fig 3A–D). Both C_3_ cropland sites (wheat and sugar beet) had similar MAE and RMSE compared with the only C_4_ cropland site (maize) which had the greatest MAE and RMSE (Fig 3C, D). C_3_ sites tended to underestimate NEE and the C_4_ site overestimated NEE.

The growing season of 2005 for the C_4_ (maize) cropland site was chosen to explore the sensitivity of the input parameters that affect the most important parameter *V*_cmax_. Increasing SLA which determines *N*_a_ could have reduced maize simulation differences from 0.93 µmol m^–2^ s^–1^ (SLA=0.08 m^–2^ g C) up to 2.25 µmol m^–2^ s^–1^ (SLA=0.1 m^–2^ g C) (Supplementary Fig. S1A). Similarly, decreasing the Flnr parameter which determines the fraction of leaf nitrogen invested in Rubisco could have reduced maize simulation differences from 0.67 µmol m^–2^ s^–1^ (Flnr=0.16) up to 1.50 µmol m^–2^ s^–1^ (Flnr=0.14) (Supplementary Fig. S1B).

### Substitution of crop Rubiscos

**Fig. 4.**
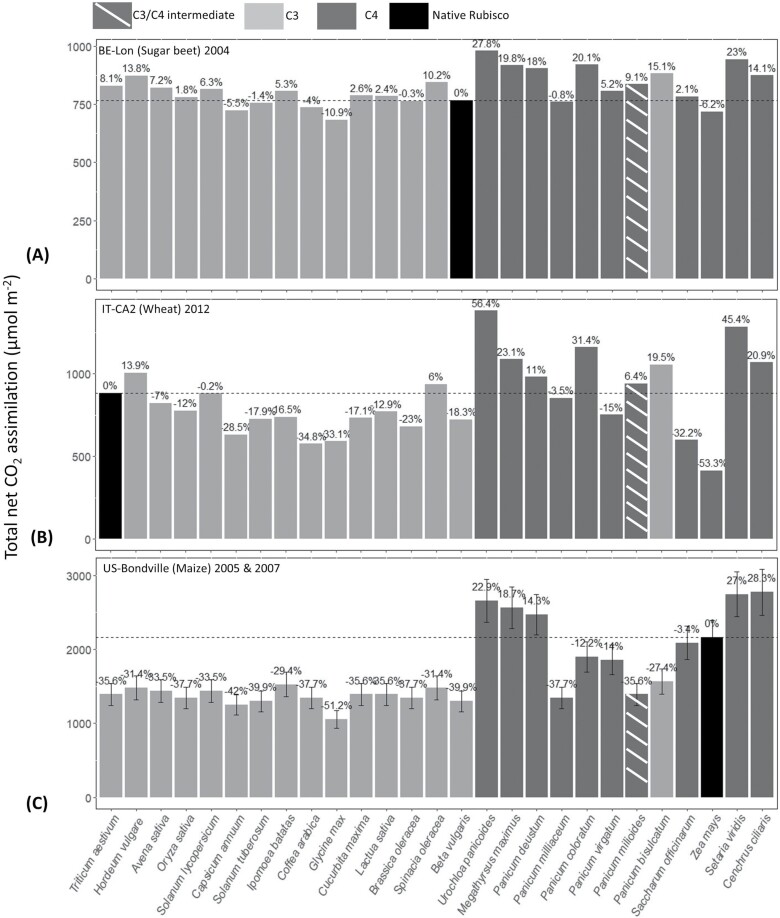
Potential total carbon uptake changes per crop growing season (µmol m^–2^). Black bars represent cropland site simulations with their native Rubiscos, and all other bars represent simulations of replacing native Rubiscos with 27 foreign Rubiscos. C_3_ plants are shown as dim grey bars, C_4_ plants as dark grey bars, and a C_3_/C_4_ intermediate patterned. Maize was given as average total carbon uptake per growing season ±SD. The total carbon uptake of crops with foreign Rubiscos are also shown as a percentage increase or decrease compared with the native Rubisco (dashed lines).

Improvements in total CO_2_ assimilation were found in all cropland sites when comparing canopy models with crop site native Rubiscos, with canopy models with foreign Rubiscos (Fig. 4).

*Triticum aestivum*, *Hordeum vulgare*, *Avena sativa*, *Oryza sativa*, *Solanum lycopersicum*, *Ipomoea batatas*, *Cucurbita maxima*, *Lactua sativa*, *Spinacia oleracea*, *Urochloa panicoide*, *Megathyrsus maximus*, *Panicum deustum*, *Panicum coloratum*, *Panicum virgatum*, *Panicum milioides*, *Panicum bisulcatum*, *Saccharum officinarum*, *Setaria viridis*, and *Cenchrus ciliaris* (19/27) Rubiscos had shown a greater total CO_2_ assimilation than the native sugar beet Rubisco (Fig. 4A). *Hordeum vulgare*, *S. oleracea*, *U. panicoides*, *M. maximus*, *P. deustum*, *P. coloratum*, *P. millioides*, *P. bisulcatum*, *S. viridis*, and *C. ciliaris* (10/27) Rubiscos had shown an overall greater total CO_2_ assimilation than the native wheat Rubisco (Fig. 4B). Maize cropland sites would benefit from substitution of only five of the foreign Rubiscos included here (Fig. 4D). These comprise *U. panicoides*, *M. maximus*, *P. deustum*, *S. viridis*, and *C. ciliaris*. Rubiscos from the Paniceae grasses were the top performing Rubiscos, with a maximum 27.8% improvement in sugar beet, 56.4% improvement in wheat, and 28.3% improvement in maize.

## Discussion

Overall, modelling species-specific Rubisco kinetics with their corresponding *H*_a_ values reproduced NEE observations reasonably well.The model simulations showed similar MAE, RMSE, and *R*^2^ to previous studies adopting ESM representations of canopy photosynthesis (Houborg *et al.*, 2009; Chen *et al.*, 2019). It is clear from the sensitivity analyses that Flnr and SLA, which determine the amount of leaf *N*_a_, are important for correctly simulating species-specific photosynthesis; this is understandable since they determine the amount of leaf *N*_a_ invested in Rubisco and thus the total photosynthesis potential of a plant (Rogers, 2014; Rogers *et al.*, 2017; Evans and Clarke, 2019). We require a framework that connects parameters such as *K*_cat_ with *N*_a_ if species-specific kinetics were to be used in ESM’s representation of photosynthesis (Wu *et al.*, 2016). Parameters that co-varied with *V*_cmax_, especially *J*_max_, may have also overestimated net CO_2_ assimilation. ESMs use the prescribed ratio of, for instance, 1.67 (*J*_max_/*V*_cmax_) and multiply this ratio by *V*_cmax_ at 25 °C to obtain a hypothetical *J*_max_ at 25 °C (Medlyn *et al.*, 2002; Kattge and Knorr, 2007). ESMs then adjust the resulting *J*_max_ for temperature using the same *H*_a_ value for all plant species. In this study, there was no alternative to using the same *H*_a_ value for the host plants as species-specific *H*_a_ measurements for *J*_max_ are hard to come by. Although it has been shown that there is little variation between the temperature response of *J*_max_/*V*_cmax_ between species, there may be some species differences in the ratio *J*_max_/*V*_cmax_ at 25 °C because of efforts to derive this ratio using different Rubisco kinetic datasets available Leuning, 2002). For example, the estimate used here—1.67—was derived by Medlyn *et al.* (2002) and another—2.00—was derived by Leuning (2002). ESMs require more consideration before using any ratio and a simpler temperature response function when *H*_a_ values are hard to come by. For instance, June *et al.* (2004) developed a simpler temperature response function that does not require *H*_a_ values and has been shown to fit numerous published datasets (Bernacchi *et al.*, 2013).

ESMs’ representations of photosynthesis also ignore limitations of mesophyll conductance imposed on CO_2_ diffusion to the site of carboxylation. It was originally assumed that the difference between CO_2_ diffusion into the stomata and chloroplast was small enough to ignore, but now it is believed that CO_2_ diffusion into the chloroplast varies with temperature and between species significantly (Von Caemmerer and Evans, 2015; Gago *et al.*, 2019; Von Caemmerer, 2020). It is important that ESMs consider incorporating such a framework for plant functional types which may help reduce model error further. There are many other species-specific considerations in ESMs which may have led to some error, but it can be assumed that most of the variation will stem from correct parameterization of Rubisco parameters as being the key rate-limiting step of photosynthesis (see review by Rogers *et al.*, 2017).

Although the simulations may never capture the pleiotropic effects of nature precisely, unlike previous Rubisco kinetic screening studies this study has utilized a state of the art canopy model to assess the performance of foreign Rubiscos in major crops, which may have been misjudged under homogenous conditions at the leaf level. For instance, previous studies failed to consider reduced carbon uptake because of interactions between the environment and stomata, and light environment, all of which could diminish carbon gains because of changes in Rubisco performance. This study reaffirms that despite all these competing factors, harnessing the natural variation of Rubisco remains among major avenues for improving plant carbon uptake.

Estimated improvements in carbon uptake with Rubisco substitutions shown here exceed previous estimates. The 56.4% improvement observed in wheat with *Urochloa panicoide* Rubisco is almost double the improvement observed by Zhu *et al.* (2004) with *Griffithsia monilis*, a non-green algae Rubisco which has one of the highest affinities for CO_2_ over O_2_ observed in nature (Zhu *et al.*, 2004; Sharwood, 2017). Improvements in crop-growing seasons were also significant with *M. maximus* and *P. deustum.* The observed improvements in wheat are probably due to the high *K*_cat_ and *S*_c/o_ of *U. panicoide*, *M. maximus*, and *P. deustum* afforded at lower temperatures; however, in warmer growing seasons (Fig. 4A, C), the improvements are lower albeit still the highest performing variants in this study (Supplementary Table S3) (Sharwood *et al.*, 2016). In contrast, *P. coloratum* and *P. bisulcatum* improvements observed in wheat and sugar beet are afforded by improvements in *S*_c/o_ but dwindle in maize because of the lower *K*_cat_ (Sharwood *et al.*, 2016). Further, when more than one growing season was available (Supplementary Fig. S2), the performance improvements are the same, suggesting the improvements would hold for the same crop at different time points (Fig. 4C).

The observed trends of transplanting other C_3_ or C_4_ crop Rubiscos are in agreement with previous findings (Hermida-Carrera *et al.*, 2016; Prins *et al.*, 2016). C_3_ crop Rubiscos such as those of *T. aestivum* and *H. vulgare* improve the performance of sugar beet in warmer growing temperatures (Fig. 4A). This is probably due to the higher *K*_cat_ with increasing temperature. C_4_ crop Rubiscos such as those of *Saccharum officinarum* perform better than C_3_ crop Rubiscos in maize due to the higher *K*_cat_ and lack of a higher *S*_c/o_ afforded by the CCM (Kubien *et al.*, 2008; Ishikawa *et al.*, 2009).

It is worth noting that there are many Rubisco variants that could also lead to dramatic improvements that were excluded from the analysis because they lacked full kinetic data. Of 217 full Rubisco kinetic sets, only 27 Rubisco species had full kinetic data with *H*_a_ values (Fig. 2) (Hermida-Carrera *et al.*, 2016; Sharwood *et al.*, 2016; Flamholz *et al.*, 2019). Indeed, there is covariation between the performance improvements of closely related species (e.g. Fig. 2, *U. panicoide* and *M. maximus*). Therefore, modelling studies will benefit from full kinetic and *H*_a_ measurements of a range of Rubisco species. For example, cold-adapted algae have similar *K*_cat_ values to C_3_ crops and higher selectivity for CO_2_ over O_2_ at low and high temperatures, which may even outperform the Paniceae Rubisco improvements in the field simulations (Young *et al.*, 2016; Iñiguez *et al.*, 2018; Valegård *et al.*, 2018). Further, Iñiguez *et al.* (2018) identified Chlorophyta Rubiscos with *K*_cat_ ranging between 4.99 and 5.08 at 25 °C. It would be valuable to measure more kinetic parameters and *H*_a_ values for Rubisco variants to enable inclusion and allow for further investigations, beyond plant species.

Although enhanced carbon uptake will be beneficial for mitigating rises in atmospheric CO_2_, it is unclear whether the fixed carbon would be transferred to the grain or edible parts of the crop (Evans and Lawson, 2020). It may be the case that additional changes in photosynthesis beyond manipulations in Rubisco traits are required to cope with the increasing carbon demand. For instance, increased *de novo* nitrogen incorporation may be required to maintain grain protein concentrations, as an inverse relationship has been shown between elevated CO_2_ and plant nitrogen (Ainsworth and Long, 2005; Evans and Clarke, 2019).

In the real-world, expressing recombinant Rubiscos in crops is still in its infancy. However, the recent progress with tobacco showed that co-expression of cognate chaperones can facilitate heterologous plant Rubisco assembly, albeit still below the wild-type level (Whitney *et al.*, 2015; Conlan *et al.*, 2019), while co-locating genes for both Rubisco large and small subunits within the chloroplast genome provides an effective bioengineering chassis for heterologous Rubisco assembly and evaluation in a whole-plant context (Martin-Avila *et al.*, 2020).

### Conclusion

This study strengthens existing Rubisco modelling studies at the leaf level by showing which Rubiscos may improve photosynthesis at the canopy level under heterogenous conditions in major cropland sites. This study also reaffirms the need for better parameterization of *V*_cmax_ with specific Rubisco/leaf nitrogen content estimates and the need for better *J*_max_ estimates at various temperatures when *H*_a_ values for species-specific *J*_max_ are difficult to come by. As more Rubisco species are surveyed, it is important that studies measure all Rubisco parameters including kinetics and heat activation values, so that these traits, and a broader diversity of Rubisco variants can be incorporated in the state of the art photosynthesis models. These models have the potential to help us further understand the diversity of Rubisco variants in nature, and which variants are worth harnessing for use in crops to maintain food supplies in future climates. They provide an invaluable tool to complement molecular and structural biology approaches in engineering Rubisco.

## Supplementary data

Fig. S1. Comparison between the original SLA and Flnr parameters and increasing SLA and decreasing Flnr increments.

Fig. S2. Potential carbon uptake changes per maize growing season.

Table S1. Model parameters.

Table S2. Measures of overall performance between modelled and observed daily net CO_2_ assimilation by maize cv. US-Bondville during growing season 2005.

Table S3. Rubisco kinetics measured at 25 °C and heat activation (*H*_a_) values used in this study.

Supplementary Protocols

erab278_suppl_Supplementary_Material

## Data Availability

The original R codes are available at: https://github.com/Iqbalwasim01/Sunlit-shaded-canopy-photosynthesis-model.git

## Abbreviations

*C*_a_: atmospheric CO_2_
*C*_c_: chloroplastic CO_2_
CCM: carbon-concentrating mechanism
*C*_i_: intercellular CO_2_
CLM: community land model
ESM: Earth system model
Flnr: fraction of leaf nitrogen invested in Rubisco
*g*_b_: leaf boundary layer conductance
GDP: growing production day
*g*_m_: mesophyll conductance
*g*_s_: stomatal conductance
*H*_a_: heat activation
*J*_max_: maximum carboxylation rate of light-limited leaf Rubisco
*K*_cat_: maximum turnover rate
*K*_c_: Michaelis–Menten constant for CO_2_
*K*_c_^21%O2^: Michaelis–Menten constant for CO_2_ at ambient atmospheric O_2_
*K*_n_: nitrogen extinction coefficient
*K*_o_: Michaelis–Menten constant for O_2_
LAI: leaf area index
MAE: mean absolute error;
*N*_a_: leaf nitrogen content
NEE: net ecosystem exchange
PAR: photosynthetically active radiation
RMSE: residual mean squared error
*S*_c/o_: specificity for CO_2_ to O_2_
SLA: specific leaf area
*V*_cmax_: maximum carboxylation rate of leaf Rubisco
