# Correction to: Rubisco substitutions predicted to enhance crop performance through carbon uptake modelling

**DOI:** 10.1093/jxb/erac295

**Published:** 2022-09-14

This is a correction to: Wasim A Iqbal, Isabel G Miller, Rebecca L Moore, Iain J Hope, Daniel Cowan-Turner, Maxim V Kapralov, Rubisco substitutions predicted to enhance crop performance through carbon uptake modelling, *Journal of Experimental Botany*, Volume 72, Issue 17, 2 September 2021, Pages 6066–6075, https://doi.org/10.1093/jxb/erab278

After being contacted by a reader, the authors have requested to amend some units and definitions in their manuscript and supplementary data file as follows:

In Figure 3 and Supplementary Figure S1, y-axis units have been corrected to “Net CO_2_ assimilation (µmol m^−2^ s^−1^)”.

In Figure 4 and Supplementary Figure S2, y-axis units have been corrected to “Seasonal net CO_2_ assimilation rate (µmol m^−2^ s^−1^ days)”. Further explanation of the calculation of the values presented in Figure 4 and Supplementary Figure S2 has been added to the legends of these figures.

In Supplementary Table S1, the definitions of some of the parameters have been corrected. These definitions have been corrected throughout the supplementary data file.

The version of record and supplementary data file have both been corrected online.

