# Supplementary material for: Rubisco substitutions predicted to enhance crop performance through carbon uptake modelling
Source: J Exp Bot. 2021 Jun 11;72(17):6066–75. doi: 10.1093/jxb/erab278 (PMC8411856; doi:10.1093/jxb/erab278)
Supplement: erab278_suppl_Supplementary_Material [file erab278_suppl_supplementary_material.pdf]

Supplementary Protocols

[1]Wasim A Iqbal, [1]Isabel Miller, [1]Rebecca Moore, [1]Iain Hope, [1]Daniel C Turner, and [1]Maxim V Kapralov

[1] School of Natural and Environmental Sciences, Newcastle University, Newcastle Upon Tyne, NE1 7RU, United Kingdom

Corresponding author: W.Iqbal@ncl.ac.uk

Corresponding author: Maxim.Kapralov@ncl.ac.uk

Model summary

The model methodology closely follows the community land surface model (CLM) 4.5 technical documentation (Bonan et al., 2013, Bonan, 2019). All constants were from the CLM unless stated otherwise (see Table S1).

Table S1. Model parameters

Symbol (Unit)	Description	Value	Reference
**Canopy temperature model**			
Rn (W m^{-2})	Net radiation of flux site for each time point	-	-
H (W m^{-2})	Sensible heat flux from flux site for each time point	-	-
λE (W m^{-2})	Latent heat flux from flux site for each time point	-	-
CL (J m^{-2} K^{-1})	Leaf heat capacity	-	- -
$T\ell$ (Kelvins)	Canopy temperature	-	
Fw	Fraction of water	0.8 (All crops)	Bonan (2019)
LMA (g m^{-2})	Leaf mass area	97.7 (C_3 crops) 87.1 (C_4 crops)	Wright et al. (2004)
Cdry (J Kg^{-2} K^{-2})	Specific heat of dry mass	1396.0 (All crops)	Bonan (2019)
Cwat (J Kg^{-2} K^{-2})	Specific heat of water	4188.0 (All crops)	Bonan (2019)
**Canopy photosynthetically active radiation model**			
$\mathrm{I}\uparrow(x)$ (W m^{-2})	Upward flux solar radiation	-	-
$\mathrm{I}\downarrow(x)$ (W m^{-2})	Downward flux solar radiation	-	-
$I^{\downarrow}_{sky,b}$ (W m^{-2})	Downward surface solar radiation from flux site for each time point	-	-
$I^{\rightarrow}b(x)$ (W m^{-2})	Direct beam radiation per leaf area	-	-
$I^{\rightarrow}d(x)$ (W m^{-2})	Diffuse beam radiation per leaf area	-	-
$I^{\rightarrow}bs(x)$ (W m^{-2})	Scatter beam radiation per leaf area	-	-

$I^{\rightarrow} sun\ (x)$ *(W m^{-2})*	Sunlit fraction diffuse radiation per leaf area	-	-
$I^{\rightarrow} shad\ (x)$ *(W m^{-2})*	Shaded fraction diffuse radiation per leaf area	-	-
$I^{\rightarrow} c\ sun$ *(W m^{-2})*	Sunlit canopy total radiation	-	-
$I^{\rightarrow} c\ shad$ *(W m^{-2})*	Shaded canopy total radiations	-	-
Kb	Direct beam extinction coefficient	-	-
$G(z)$	Goudriann function	-	Goudriaan (1988)
τd	Diffuse transmittance from sky hemisphere	-	-
Kd	Diffuse beam extinction coefficient	-	-
$a_s(\mu)$	Single scattering albedo	-	-
$\omega\ell$	Scattering coefficient (visible)	-	-
$\beta\omega\ell$	Diffuse beam upward scatter parameter	-	-
$\beta_0\omega\ell$	Direct beam upward scatter parameter	-	-
$\cos y\ell$ or Lh	Leaf area projected in the direction of the solar beam	-	-
$\Theta\ell$	Leaf inclination angle	-	-
τd	Transmittance	-	-
$P\ell$	Leaf reflectance (visible)	0.11 (All crops)	CLM 4.5
$\tau\ell$	Leaf transmittance (visible)	0.05 (All crops)	CLM 4.5
Pgd	Soil albedo	0.01 (All sites)	CLM 4.5
$x\ell$	Ross-index	-0.3 (Sugar beet)<br>-0.65 (Wheat)<br>-0.5 (Soybean)<br>-0.5 (Maize)	CLM 4.5
**Photosynthesis and CO_2 diffusion model**			

An ($\mu mol\ CO_2\ m^{-2}\ s^{-1}$)	Net CO_2 assimilation	-	-
Ac ($\mu mol\ CO_2\ m^{-2}\ s^{-1}$)	RuBP carboxylase (Rubisco) limited rate	-	-
Aj ($\mu mol\ CO_2\ m^{-2}\ s^{-1}$)	Light limited rate	-	-
Ap ($\mu mol\ CO_2\ m^{-2}\ s^{-1}$)	Phosphate limited rate for C3 plants or phosphoenolpyruvate carboxylase limited rate for C4 plants	-	-
$Vcmax$ ($\mu mol\ CO_2\ m^{-2}\ s^{-1}$)	Maximum carboxylation rate of leaf Rubisco	-	-
$Jmax$ ($\mu mol\ CO_2\ m^{-2}\ s^{-1}$)	Light limited maximum carboxylation rate of Rubisco for C_3 plants	$1.67\ Vcmax$	CLM 4.5
Sc/o	Specificity of Rubisco for CO_2 compared to O_2	-	-
Kc (μM)	Rubisco Michaelis Menten constant for CO_2	-	-
Ko ($mmol\ 0_2\ mol^{-1}$)	Rubisco Michaelis Menten constant for O_2	278.4 (All crops)	CLM 4.5
Oi ($mmol\ 0_2\ mol^{-1}$)	Intercellular O_2	210 (All crops)	CLM 4.5
Ci ($\mu mol\ C0_2\ mol^{-1}$)	Intercellular CO_2	-	-
$\Gamma *$	CO_2 compensation point	-	-
Tp ($\mu mol\ C0_2\ mol^{-1}$)	Triose phosphate utilization for C_3 plants	$0.167\ Vcmax$	CLM 4.5
Kp ($\mu mol\ C0_2\ mol^{-1}$)	Phosphoenylpyruvate carboxylase limited rate of net CO_2 assimilation	$20000\ Vcmax$ (C_4)	CLM 4.5
Kd ($\mu mol\ C0_2\ mol^{-1}$)	Loss of gross CO_2 to dark respiration.	$0.015\ Vcmax$ (C_3 crops)<br>$0.025\ Vcmax$ (C_4 crops)	CLM 4.5
ϕ ($\mu mol\ photon\ m^{-2}$)	Absorbed canopy photosynthetic active radiation for	$I^{\rightarrow}sun4.6$ or $I^{\rightarrow}shad4.6$	-

	shaded or sunlit fraction		
a (mol CO_2 mol^{-1})	Quantum efficiency for C_4 plants	0.05	CLM 4.5
$\Theta PSII$	Smooths transition of $Jmax$ in response to changes in ϕ.	0.7	CLM 4.5
$\Phi PSII$	Quantum yield of photosystem II	0.85	CLM 4.5
Ai (µmol CO_2 m^{-2} s^{-1})	Intermediate rate of Ac and Aj rates for smoothing transition to Ap rate	-	-
Θ_{cj}	Smoothing parameters	-	-
Θ_{ip}	Smoothing parameters	-	-
A (µmol CO_2 m^{-2} s^{-1})	Gross CO_2 assimilation	-	-
Ca (µmol CO_2 mol^{-1})	Ambient atmospheric CO_2	360 (All sites)	-
Cc (µmol CO_2 mol^{-1})	Chloroplast CO_2	-	-
Cs (µmol CO_2 mol^{-1})	Surface CO_2	-	-
Gb (mol H_2O mol^{-1})	Leaf boundary layer conductance	-	-
Gs (mol H_2O mol^{-1})	Stomatal conductance	-	-
Gm (mol H_2O mol^{-1})	Mesophyll conductance	-	-
$g0$ (mol H_20 mol)	Minimum stomatal conductance	0.01 (All crops)	CLM 4.5
$g1$ (mol H_20 mol)	Maximum stomatal conductance	5.25 (C_3 crops)<br>1.62 (C_4 crops)<br>1.79 (Maize)<br>5.79 (Wheat)<br>5.79 (Rice)<br>5.79 (Soybean)	CLM 4.5
hs	Relative humidity	-	-
Ci/Ca	Ca to Ci conversion ratio	0.67 (C_4)<br>0.87 (C_3)	Bonan (2019)
bw	Soil moisture stress factor	-	-
θ	Soil moisture content	-	-

θ_w	Field wilting point	0.1 (All cropland sites)	Bonan (2019)
θ_c	Saturated soil moisture	0.477 Silty clay loam (All cropland sites)	Campbell (1974)
**Temperature adjustments**			
$Param25$ (Kelvin)	Rubisco kinetic parameter measured at 25°C	-	-
$ParamT$ (Kelvins)	Temperature adjusted kinetic parameter	-	-
$Param.c3$ or $Param.c4$ (Kelvins)	Temperature adjusted kinetic parameter adjusted for extreme temperatures.	-	-
T (Kelvins)	Canopy temperature	-	-
Ha (*KJ mol^{-1}*)	Heat activation for Rubisco kinetics	-	-
$Ko25\ Ha$ (*KJ mol^{-1}*)	Heat activation for Rubisco Michaelis Menten constant for O_2 at 25°C	35.94 (All crops)	CLM 4.5
$R\ gas$ *(J mol K^{-1})*	Universal gas constant	8.314	-
Hd (*KJ mol^{-1}*)	Heat deactivation for Rubisco kinetics	200 (All crops both Vcmax and Jmax)	CLM 4.5
ΔS (*KJ mol^{-1}*)	Entropy term	0.66839 (All crops Vcmax)<br>0.65970 (All crops Jmax)	CLM 4.5
$s1$ (*Kelvins*)	-	0.3	CLM 4.5
$s2$ (*Kelvins*)	-	313.15	
$s3$ (*Kelvins*)	-	0.2	
$s4$ (*Kelvins*)	-	288.15	-
**Gradient of Rubisco kinetics throughout the canopy**			
$Kcat25$ *(s^{-1})*	Maximum carboxylation catalytic rate of Rubisco	-	-
$Vcmax25$ *(μmol m^{-2} s^{-1})*	Maximum carboxylation rate of leaf Rubisco at 25°C	-	-

$Vcmax25$,sun *(μmol m^-2 s^-1)*	Maximum rate of sunlit canopy Rubisco at 25^{0}C	-	-
$Vcmax25$,shad *(μmol m^-2 s^-1*	Maximum rate of shaded canopy Rubisco at 25^{0}C	-	-
$FLNR$	Fraction of leaf nitrogen in Rubisco	0.412 (Wheat)<br>0.412 (Sugar beet)<br>0.412 (Soybean)<br>0.1758 (Maize)	CLM 4.5
SLA *(g C m^-2)*	Specific leaf area: carbon content per unit leaf area at the top of the canopy	0.07 (All crops)	CLM 4.5
N *(g N m^-2)*	Nitrogen content per unit leaf area at the top of the canopy	-	-
$C:N$	Carbon to nitrogen ratio	25 (All crops)	CLM 4.5
$C0$	*Conversion factor for moles of Rubisco catalytic sites*	7/5500	Houborg et al. (2013)

1 Canopy temperature

Leaf temperature is solved using surface fluxes of sensible heat flux H, latent heat flux λE and net radiation Rn at each time point (Bonan, 2019).

$$CL\frac{\partial T\ell}{\partial t} = Rn - H - \lambda E \qquad (1)$$

Where H and λE are subtracted from the total net radiation Rn of the site surface. The remaining energy retained by the surface is represented using a leaf heat capacity CL. CL is a function of leaf mass per unit area LMA of a leaf. The fraction of ma which is water is assumed (e.g. 80% in crop species) fw and the fraction of LMA which is dry matter is assumed to be $1 - fw$. Heat capacity constants of dry matter $Cdry$ and water $Cwat$ are also assumed for the dry and water fractions. CL is represented by the following equation:

$$CL = \frac{LMA}{1 - fw}[(1 - fw)Cdry + fwCwat] \quad (2)$$

2 Canopy photosynthetically active radiation

Liou (2002) provided an analytical solution for the two-stream approximation as described by Bonan (2019), (Bonan et al., 2011).

$$\mathrm{I}\uparrow(\mathrm{x}) = -y_1 e^{-Kb\Delta L} + n_1 u\, e^{-h\Delta L} + n_2 v e^{h\Delta L} \quad (3)$$

$$\mathrm{I}\downarrow(\mathrm{x}) = -y_2 e^{-Kb\Delta L} + n_1 v\, e^{-h\Delta L} + n_2 u e^{h\Delta L} \quad (4)$$

Equation (3) gives the upward flux of direct beam solar radiation above the leaves and equation (4) gives the downward flux below the leaves at depth equal to the cumulative leaf area index; ΔL =LAI. In this study, clumping index was ignored (Ω=1). Inputs for equations (3) and (4) are:

$$\mathrm{b} = [1-(1-\beta)\omega\ell]\mathrm{Kd}, \quad \mathrm{c} = \beta\omega\ell\mathrm{Kd} \quad (5)$$

$$\mathrm{h} = \sqrt{b^2 - c^2} \quad (6)$$

$$\mathrm{u} = \frac{h-b-c}{2h}, \quad v = \frac{h+b+c}{2h} \quad (7)$$

$$y_1 = [\beta_0 Kb - b\beta_0 - c(1-\beta_0)]\frac{\omega\ell\mathrm{Kb}I^{\downarrow}_{sky,b}}{h^2 - Kb^2} \quad (8)$$

$$y_2 = \left[(1-\beta_0)\, Kb + c\beta_0 + b(1-\beta_0)\right]\frac{\omega\ell\mathrm{Kb}I^{\downarrow}_{sky,b}}{h^2 - Kb^2} \quad (9)$$

$$n_1 = \frac{y_2 - n_2 u}{v} \quad (10)$$

$$n_2 = \frac{\mathrm{v}\left(y_1 + y_2 P_{gd} + P_{gd} I^{\downarrow}_{sky,b}\right)e^{-KbLAI} - y_2(u + vP_{gd})e^{-hLAI}}{v\left(v + uP_{gd}\right)e^{hLAI} - u\left(u + vP_{gd}\right)e^{-hLAI}} \quad (11)$$

$I^{\downarrow}_{sky,b}$ is the total downward direct beam solar radiation flux which can be obtained from eddy covariance data sets for each site. $\omega\ell = \mathrm{P}\ell + \tau\ell$ is a scattering beam coefficient obtained by adding a known leaf reflectance $\mathrm{P}\ell$ and transmittance $\tau\ell$. P_{gd} is a known soil albedo. The proportion of direct (β_0 and diffuse beam β radiation scattered is then obtained using the following equations as described by Sellers (1985):

$$\beta\omega\ell = \left[\frac{1}{2}\,[\,\mathrm{P}\ell + \tau\ell + (\mathrm{P}\ell - \tau\ell\,)\cos 2\Theta\ell\right] \quad (12)$$

$$\beta_0\omega\ell = \left[\frac{Kb + Kd}{Kb}\right] a_s(\mu) \quad (13)$$

$\Theta\ell$ is the mean leaf inclination angle. $\cos 2\Theta\ell$ was approximated using the Ross-index $\chi\ell$ which describes the departure of leaf angle from a spherical distribution. For crops $\chi\ell$ was obtained from the CLM4.5 as follows:

$$\cos 2\Theta\ell = \left(\frac{1+\chi\ell}{2}\right)^2 \quad (14)$$

The coefficient Kb which is the direct beam extinction coefficient (defined as the optical depth per unit leaf area) is probably one of the most important parameters in canopy modelling as it

determines the sunlit and shaded fraction of the canopy models. Kb is defined by the following equation:

$$Kb = \frac{Lh}{L} = \frac{\cos y\ell}{\cos SZA} = \frac{G(Z)}{\cos SZA} \qquad (15)$$

$\cos y\ell$ or $G(Z)$ or Lh is the amount of leaf area projected in the direction of the solar beam. An approximate for $\cos y\ell$ or Lh is obtained using the Goudriaan (1988) function $G(Z)$ and the corresponding $\chi\ell$ as follows:

$$G(Z) = \emptyset 1 + \emptyset 2 \cos SZA \qquad (16)$$

The coefficients for equation 16 are:

$$\emptyset 1 = 0.5 - 0.633\chi\ell - 0.33\chi\ell^2, \qquad \emptyset 2 = 0.877(1 - 2\emptyset 1) \qquad (17)$$

$\cos SZA$ adjusts $\cos y\ell$ or $G(Z)$ or Lh onto a horizontal plane. $\cos SZA$ is obtained from the solar zenith angle SZA which can easily be obtained from remote sensing data or established relationships based on location (latitude and longitude) and time (i.e. time zone month, year, hour day). In this study, SZA for each half hourly observation was approximated using the 'fishmethods' R package which adopts analytical equations by Frouin et al. (1989).

Because diffuse radiation is assumed to come from all parts of the sky (unlike direct beam radiation), the extinction coefficient for direct beam radiation Kd was approximated by dividing the sky hemisphere into three 30° increments and summing the transmittance obtained from these three angles to give an approximate of diffuse transmittance as follows:

$$\tau d = 2 \sum_{i=1}^{3} \exp\left(-\frac{G(Zi)}{\cos SZA}\right) \sin SZA \cos SZA \,\Delta Zi \qquad (18)$$

Where $\frac{G(Zi)}{\cos SZA}$ is the Kb coefficient for each 30° increment obtained using equation (15). Using the trigonometric relationship $2 \sin SZA \cos SZA \,\Delta Zi$ gives the total contribution of each angle to total sky irradiance. Each 30° increment of the sky hemisphere was assumed to be equally spaced apart (i.e. ΔZi=0.1745 radians) (Bonan, 2019). The final Kd coefficient for diffuse radiation is given using the following equation:

$$Kd = -\frac{ln \ \tau d}{LAI} \qquad (19)$$

Finally, the single scattering albedo $a_s(\mu)$ for equation (13) was obtained using the following equation:

$$a_s(\mu) = \left(\frac{\omega\ell}{2}\right) \frac{G(Z)}{G(Z) + cosSZA\emptyset 2} \left\{1 - \frac{cosSZA\emptyset 1}{G(z) + cosSZA\emptyset 2} ln\left[\frac{G(z) + cosSZA\emptyset 1 + cosSZA\emptyset 2}{cosSZA\emptyset 1}\right]\right\} \ (20)$$

Where $G(Z)$, Ø2 and Ø1 are obtained from the Goudriaan (1988) function (16).

The overall radiative balance for direct beam radiation is given by considering the upward downward flux (per leaf area) at a depth equal to the cumulative LAI x:

$$I^{\rightarrow}b(x) = I_b^{\uparrow}(LAI) - I_b^{\downarrow}(LAI) \qquad (21)$$

Similar equations pertain for diffuse beam radiation $I^{\rightarrow}d(x)$ but involve dropping direct beam terms in equation (3) and (4) and changing coefficients n_1 and n_2 (see pg.252 Bonan (2019)).

The sum of the diffuse $I^{\rightarrow}d(x)$ and scattered beam radiation $I^{\rightarrow}bs(x)$ gives the radiation absorbed by the shaded leaves $I^{\rightarrow}sha$ (per shaded leaf area) at the cumulative leaf area index x:

$$I^{\rightarrow}sha(x) = I^{\rightarrow}d(x) + I^{\rightarrow}bs(x) \qquad (22)$$

Where the $I^{\rightarrow}bs(x)$ is:

$$I^{\rightarrow}bs(x) = \omega\ell \mathrm{Kb} I^{\downarrow}_{sky,b} e^{-KbLAI} + I^{\rightarrow}b(x) \qquad (23)$$

The shaded radiation absorbed by sunlit leaves (per sunlit leaf area) is:

$$I^{\rightarrow}sun(x) = I^{\rightarrow}sha(x) + (1 - \omega\ell)\mathrm{Kb}\, I^{\downarrow}_{sky,b} \quad (24)$$

Integrated over the sunlit $LAIsun$ and shaded fraction of the canopy $LAIshad$:

$$I^{\rightarrow}c\ shad = I^{\rightarrow}shad(x) \cdot LAIshad \ (25)$$

$$I^{\rightarrow}c\ sun = I^{\rightarrow}sun(x) \cdot LAIsun \ (26)$$

The sunlit and shaded LAI fractions are given as follows:

$$LAIsun = \frac{1 - e^{(-KbLAI)}}{Kb} \ (27)$$

$$LAIshad = LAI - LAIsun \ (28)$$

3 Photosynthesis model and CO_2 diffusion

The Farquhar et al. (1980) model for C_3 photosynthesis and Collatz et al. (1992) model for C_4 photosynthesis were implemented in this study. Both models depend on a set of parameters (Table S1). Briefly, leaf net photosynthesis An is the minimum of RuBP carboxylate (Rubisco) limited rate Ac , light limited rate Aj and the phosphate limited rate Ap for C_3 photosynthesis or phosphoenolpyruvate (PEP) carboxylase limited rate for C_4 photosynthesis minus dark respiration Rd (29).

$$An = \min(Ac, Aj, Ap) - Rd \quad (29)$$

The RuBP carboxylate limited rate Ac is given using the hyperbolic function for C_3 photosynthesis and $Vcmax$ for C_4:

$$Ac = \begin{cases} \dfrac{Vcmax(ci - \Gamma *)}{Ci + Kc(1 + \frac{Oi}{Ko})} & C3\,plants \\ Vcmax & C4\,plants \end{cases} \quad (30)$$

The light limited rate Aj is given using another hyperbolic function for C_3 photosynthesis and in relation to quantum efficiency for C_4:

$$Aj = \begin{cases} \dfrac{Jmax(ci - \Gamma *)}{4ci + 8\Gamma *} & C3\,plants \\ a(4.6)\phi & C4\,plants \end{cases} \quad (31)$$

The phosphate limited rate Ap is given for C_3 and PEP carboxylase limited rate for C_4 plants as follows:

$$Ap = \begin{cases} 3Tp & C3\,plants \\ KpCi & C4\,plants \end{cases} \quad (32)$$

In equations 30-31, $Vcmax$, $Jmax$, Sc/o, Kc and Ko are the Michaelis-Menten kinetics for the maximum velocity of Rubisco $Vcmax$ and its corresponding light limited rate $Jmax$, specificity for carbon dioxide (CO_2) to oxygen (O_2) Sc/o, Michaelis-Menten constant for CO_2 Kc, and Michaelis-Menten constant for O_2 Ko, respectively. Ci is the amount of atmospheric CO_2 Ca that is assumed to be in the leaf. Oi is the amount of atmospheric oxygen assumed to be present in the leaf. For simplicity, this is kept constant at $Oi = 210\,mmol\,O_2\,mol^{-1}$. $\Gamma *$ is the CO_2 compensation point given as $\Gamma * = 0.5(\frac{Oi}{Sc/o})$. The quantum efficiency a for C_4 plants is assumed to be $a = 0.05\,mol\,CO_2\,mol^{-1}\,photon$. ϕ is the absorbed PAR which is obtained from equation 27 and 28 for sunlit and shaded fractions of the canopy. The triose phosphate utilization rate Tp is obtained using the relationship: $Tp = 0.167Vcmax$. Similarly, the initial slope of the CO_2 response curve for PEP carboxylase limited rate Kp for C_4 plants is given: $Kp = 20000Vcmax$.

For C_3 plants $Jmax$ is obtained using the relationship: $Jmax = 1.65Vcmax$. Jmax is also dependent on PAR so requires adjustment for variations in PAR. In the CLM, this is given using the quadratic function and the solution is the smaller root:

$$\Theta PSIIJmax^2 - (\mathrm{I}PSII + Jmax)Jmax + IPSIIJmax = 0 \quad (33)$$

$\Theta PSII$ is a curvature parameter which is set as 0.7 in this study. $\mathrm{I}PSII$ is the amount of light used in photosystem II given as follows:

$$\mathrm{IPSII} = 0.5\Phi PSII\phi \quad (34)$$

$\Phi PSII$ is the quantum yield of photosystem II which is assumed to be $\Phi PSII = 0.85$ and 0.5 partitions photons between both photosystems.

In this study, the co-limitation as described by Collatz et al. (1992) is used which smooths the transition between $Ac, Aj\ and\ Ap$ using the following quadratic functions:

$$\Theta_{cj} A_i^2 - (A_c + A_j)A_i + A_c A_j = 0 \quad (35)$$

$$\Theta_{ip} A - (A_i + A_p)A + A_i A_p = 0$$

The first quadratic function in equation (35) obtains an intermediate gross CO_2 assimilation A_i (i.e. between A_c and A_j rates) and then A_i and A_p are used to obtain the final gross CO_2 assimilation. Θ_{cj} and Θ_{ip} are smoothing parameters for C_3 or C_4 plants.

4. Temperature adjustments for Rubisco kinetics

The Rubisco kinetic parameters $Vcmax$, $Jmax$, $\Gamma *$, Kc and Ko all depend on temperature. The kinetics measured at 25°C were adjusted using the Arrhenius equation (Bonan, 2019, Bonan et al., 2011).

$$ParamT = Param25 \cdot e^{\left[\frac{Ha}{298.15 \cdot 0.001 \cdot Rgas} \cdot \left(1 - \frac{298.15}{T}\right)\right]} \quad (36)$$

Ha is the species-specific heat activation value (KJ mol^{-1}), $Rgas$ is the universal gas constant, T is the canopy temperature obtained from equation (1) and $Param25$ is a species-specific kinetic parameter at 25°C. $ParamT$ is the temperature adjusted kinetic parameter. Since some parameters (e.g. Rd, $Jmax$, and Kp) vary with $Vcmax$ for simplicity some parameters were adjusted according to temperature changes of $Vcmax$ except Jmax. It is understood that the temperature response of Jmax is closely related to the lipid composition and thylakoid composition (Von Caemmerer, 2000). For simplicity, an average C3 Ha value was used for all crop species Jmax.

The temperature adjusted parameters were additionally adjusted for enzyme breakdown at extreme temperatures. Separate temperature functions with constants for C_3 and C_4 crops are used as it is unknown whether there are significant inter-species differences.

For C_3 photosynthesis the temperature deactivation function is:

$$Param.c3 = ParamT \cdot \frac{1 + e^{\left(\frac{298.15\Delta S - Hd}{298.15 \cdot 0.001 \cdot Rgas}\right)}}{1 + e^{\left(\frac{\Delta S T - Hd}{0.001 \cdot Rgas \cdot T}\right)}} \quad (37)$$

Where ΔS (KJ mol^{-1}) is an entropy term and Hd is the heat deactivation value (KJ mol^{-1}) (Table S1).

For C_4 photosynthesis the temperature deactivation function is (Sellers et al., 1992, Collatz et al., 1992):

$$Param.c4 = ParamT \cdot \left[1 + e^{(s1(T - s2))}\right] \cdot \left[1 + e^{(s3(s4 - T))}\right] (38)$$

Where the parameters $s1, s2, s3, s4$ (K^{-1}) adjust the rate of increase/decrease of $ParamT$ at low or high temperatures.

5. Coupling photosynthesis models to CO_2 diffusion model

Both photosynthesis models must be coupled to a model which quantifies the amount of Ca that reaches the site of carboxylation in the leaf. Mathematical expression for full CO_2 diffusion from the atmosphere to the site of carboxylation which is in the chloroplast Cc is as follows:

$$Cc = Ca - \frac{A}{Gb} - \frac{A}{Gs} - \frac{A}{Gm} \quad (39)$$

A is the gross photosynthesis assimilation and Gb, Gs, and Gm are the CO_2 conductance of the leaf boundary layer, stomata and mesophyll, respectively given as mol H_2O m^{-2} s^{-1} . In Earth system models (ESMs) CO_2 diffusion into the mesophyll Gm is often ignored as it remains uncertain how to correctly parameterise Gm in ESMs (Rogers et al., 2017). Gm is dropped from equation (27) and the intercellular CO_2 Ci is assumed to be a product of Gb and Gs as follows:

$$Ci = Ca - \frac{1.4}{Gb}A - \frac{1.6}{Gs}A \quad (40)$$

In equation (39) Gb and Gs are calculated for H_2O and 1.4 and 1.6 in equation (40) adjusts the conductance for H_2O to that of CO_2 which accounts for the lower diffusivity of CO_2 compared to H_2O.

In this study Gs was calculated using the semi-empirical Ball & Berry stomatal model as follows (Bonan, 2019, Franks et al., 2017):

$$Gs = g0 + g1bw\frac{A}{Cs}hs \quad (41)$$

The Ball & Berry model assumes that stomatal conductance varies linearly with A, relative humidity hs and surface CO_2 Cs. $g0$ and $g1$ are known minimum and maximum Gs values. bw is a soil moisture stress parameter which adjusts the relationship for poorly watered conditions. This is required since $g0$ and $g1$ measurements are often done in well-watered conditions. For simplicity it was assumed that leaf hs was the same as surface humidity reported by the flux data sets. Cs is obtained from Gb using equations derived from engineering studies as follows (see p.159 Bonan (2019)):

$$Gb = a(\frac{u}{\ell})^{0.5} \quad (42)$$

a and u are parameters that describe the effect of wind speed on a leaf's length ℓ. In this study, ℓ was assumed to be 0.05 meters.

bw in equation (41) was calculated using the reported soil water content θ for each half hourly observation for each site as follows:

$$bw = \begin{cases} 1 & \theta \geq \theta_c \\ \frac{\theta - \theta_w}{\theta_c - \theta_w} & \theta_w < \theta < \theta_c \\ 0 & \theta \leq \theta_w \end{cases} \quad (43)$$

θ_w is the field wilting point which is assumed to be 0.1 (or 10%). θ_c is when soil moisture is assumed to be saturated. In this study, θ_c values for different soil textures were derived from Campbell (1974). θ is the soil water content from each half hourly observation.

Because equation (40) is also dependent on photosynthesis A and the solution to equation (40) Ci is also an input into the photosynthesis models. Equation (40) requires a numerical approach. In this study, the Newton-Raphson root finding method was used as described by Sun et al. (2012). The Newton-Raphson approach is as follows:

$$x_{n+1} = x_n - \frac{f(x_n)}{f'(x_n)} \qquad (44)$$

Sun et al. (2012) solved equation (44) by treating equation (40) as a transcendent function which is in the form $f(x) = h(x) - g(x)$. $f(x)$ is the function to be zeroed, $h(x)$ is the stomatal dependent CO_2 diffusion equation and $g(x)$ is the function to obtain the initial guess for Ci. The initial guess for Ci is obtained using a ratio of Ca to Ci conversion for C_3 and C_4 plants. The final iteration function is:

$$f(x) = \left[Cs - \frac{A(Ci)}{Gs}\right] - [(Ci/Ca)Ca] \qquad (45)$$

Newton Raphson method requires the derivative $f'(x)$ of $f(x)$. Sun et al. (2012) used a simple numerical approximate using a finite difference:

$$f'(x) = \frac{[f(x + f(x)) - f(x)]}{f(x)} \qquad (46)$$

This initial guess is passed through a series of equations:

$$Ci = (Ci/Ca)Ca \qquad (47)$$

$$A = \min\,(Ac(Ci), Aj(Ci), Ap) \qquad (48)$$

$$Cs = Ca - \frac{A}{Gb} \qquad (49)$$

$$0 = Gs^2 + \left[Gb - g0 - \frac{g1Abw}{Cs}\right]Gs - Gb\left[g0 + \frac{g1Abw}{Cs}\frac{e_a}{e_{sat}}\right] \qquad (50)$$

$$Ci = Cs - \frac{A}{Gs} \qquad (51)$$

The full iteration scheme was as follows:

1. An initial guess for Ci was obtained (47).
2. A was obtained using equation (48).
3. Cs was obtained using equation (49).
4. Gs was obtained using a quadratic function of the Ball & Barry equation (50).
5. Final Ci was obtained using equation (51).
6. The iterative function $f(x) = h(x) - g(x)$ (45) was formed using the initial Ci guess from step 1 and final Ci guess from step 5.
7. A finite difference for the derivative $f'(x)$ was obtained by adding the initial Ci and final Ci.
8. The finite difference was then used to obtain an intermediate A, Cs, Gs and final Ci (48-51).
9. An approximate of the derivative was obtained according to equation (46) as follows; An intermediate iteration function (45) was obtained from subtracting initial Ci (from step 1) and final intermediate Ci (from step 8). Then, the intermediate iteration function formed the numerator in equation (46) and iteration function from step 6 formed the denominator.

10. Steps 2-9 were repeated until the convergence criteria was met (<0.001 µmol CO_2) and the upper limit of iterations was set to N= 20.

If the upper limit was reached because convergence was not met, the bisection method was used which guarantees convergence (Burden, 2016). The bisection method continuously bisects an interval containing the correct solution 'x' until convergence is achieved.

For each iteration, the bisection method is in the form of:

$$c = \frac{a+b}{2} \quad (52)$$

c is the midpoint of the interval a and b. The full iteration scheme was as follows:

1. To obtain the first c guess, a, the lower interval was assumed as the lowest possible Ci that could be achieved; $\Gamma *$. b was assumed as the highest Ci that could theoretically be achieved: Ca (52).
2. An initial A was obtained using the initial c guess (48).
3. The initial A was used to obtain an initial Cs (49).
4. The initial Cs was used to obtain an initial Gs using a quadratic function of the Ball and Berry equation (50).
5. The initial Gs was used to obtain an initial Ci (51).
6. If the difference $Ci - c$ was below the convergence criteria (<0.001 µmol CO_2) then the iteration stopped, and convergence was met.
7. If the convergence criteria were not met, bracketing of the interval continued as follows: the initial $'a'$ was re-calculated using the Ci function (48-51) to obtain $Ci(a)$ and then the difference $Ci(a) - c$. If the sign of the difference $Ci - c$ and the difference $Ci(a) - c$ were positive, the initial midpoint c would become the next iteration a otherwise if either of the signs were negative the initial a remained unchanged. Similarly, if the sign of the difference $Ci - c$ and the difference $Ci(a) - c$ were positive, the initial c midpoint would become the next iteration $'b'$, or remain unchanged.
8. Finally, if the signs of the initial difference $Ci - c$ and the difference $Ci(a) - c$ were positive the next iteration's difference $Ci(a) - c$ would become $Ci - c$ otherwise remain unchanged. Steps 2 to 8 were repeated until the convergence criteria was met.

6. Gradient of Rubisco kinetics throughout the canopy

Rubisco kinetics including the Sc/o, Kc and Ko were assumed to be the same throughout the canopy. An approximate of $Vcmax25$ measured at 25°C for the top of the canopy was obtained as described by Houborg et al. (2013):

$$Vcmax25 = C0 \cdot Kcat25 \cdot Flnr \cdot N \cdot 10^6 \qquad (53)$$

$Kcat25$ is the maximum catalytic rate of Rubisco measured at 25°C, $Flnr$ is the fraction of leaf nitrogen in Rubisco, N leaf nitrogen content and $C0$ is a conversion factor. N was obtained as follows:

$$N = \frac{1}{C{:}N \cdot SLAtop} \qquad (54)$$

$C{:}N$ is a carbon to nitrogen ratio. $SLAtop$ is the specific leaf area at the top of the canopy.

$Vcmax25$ for the sunlit and shaded fractions of the canopy was obtained as follows (Bonan, 2019, Bonan et al., 2011):

$$Vcmax25, sun = Vcmax25 \cdot \left\{ \left[1 - e^{-(Kn+Kb)LAI}\right] \frac{1}{Kn+Kb} \right\} \quad (55)$$

$$Vcmax25, shad = Vcmax25 \cdot \left\{ \left[1 - e^{-Kn\, LAI}\right] \frac{1}{Kn} - \left[1 - e^{-(Kn+Kb)LAI}\right] \frac{1}{Kn+Kb} \right\} \quad (56)$$

Kb obtained from equation (19) and the nitrogen coefficient that describes the vertical gradient of nitrogen Kn was assumed to be the same for all crops (Kn=0.3).

7. Sensitivity of Vcmax parameters to model performance

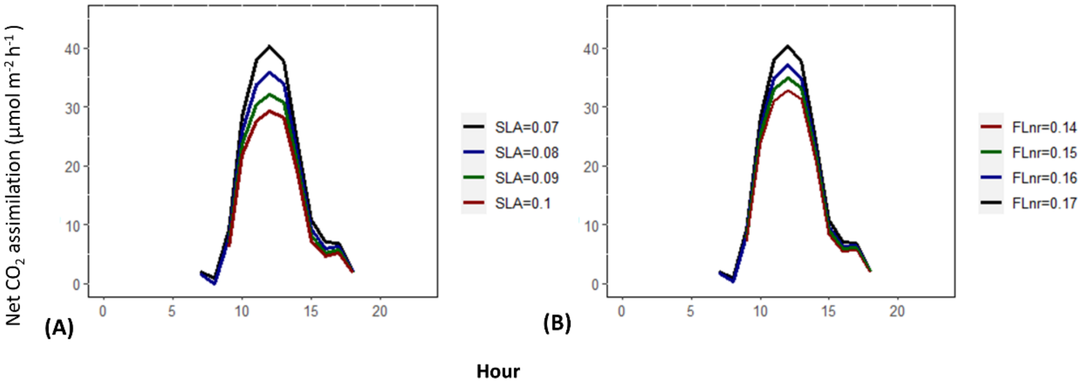

Figure S1. Comparison between the original SLA and Flnr parameters (black) and increasing SLA (A) and decreasing Flnr increments (B). Plots represent a single average diurnal period of the US-Bondville 2005 growing season. SLA and Flnr are both crucial parameters for estimating Vcmax in the canopy models. The SLA gives the leaf Na (see equation 53): The total leaf Vcmax is given using the leaf Na and FLnr. Leaf Vcmax is then scaled to the canopy (see supplementary equation 54).

8. Modelling seasonal leaf area index

LAI is a major driving force of the simulations (Bonan, 1993). The CLM uses a complex carbon-nitrogen biogeochemical model to predict seasonal LAI prospectively. Since the aim in this study was to retrospectively model changes in photosynthesis, there are less complicated methods for obtaining seasonal trends of LAI for retrospective analysis.

To rule out any errors in the simulations arising from inaccurate LAI estimates, three LAI retrieval methods were tested for their ability to accurately simulate flux site net CO_2 assimilation trends retrospectively. The three LAI methods included a normalised vegetation index (NDVI) method derived from flux tower incoming and outgoing photosynthetically active radiation (see Wilson and Meyers (2007)), eight day MODIS satellite LAI product (https://modis.ornl.gov/sites/) and the growing production day (GPD) method which uses estimates of gross primary productivity (GPP) as a proxy of canopy size but modified here to use tower derived GPP rather than estimates from the MODIS GPP algorithm (see Xin et al. (2019)). 2005 growing season of the US-Bondville maize site was chosen for the simulations.

Table S2. Measures of overall performance between modelled and observed daily net CO_2 assimilation by maize cv. US-Bondville during growing season 2005. MAE is the mean absolute error, RMSE is the residual mean squared error, R^2 is the coefficient of determination. RMSE and MAE have units of μmol CO_2 m^{-2} day^{-1}

LAI	MAE	RMSE	R^2
MODIS	8.49	10.60	0.76
NDVI	11.23	14.11	0.77
GPD	5.74	7.52	0.90

9. Growing season improvements of the US-Bondville site

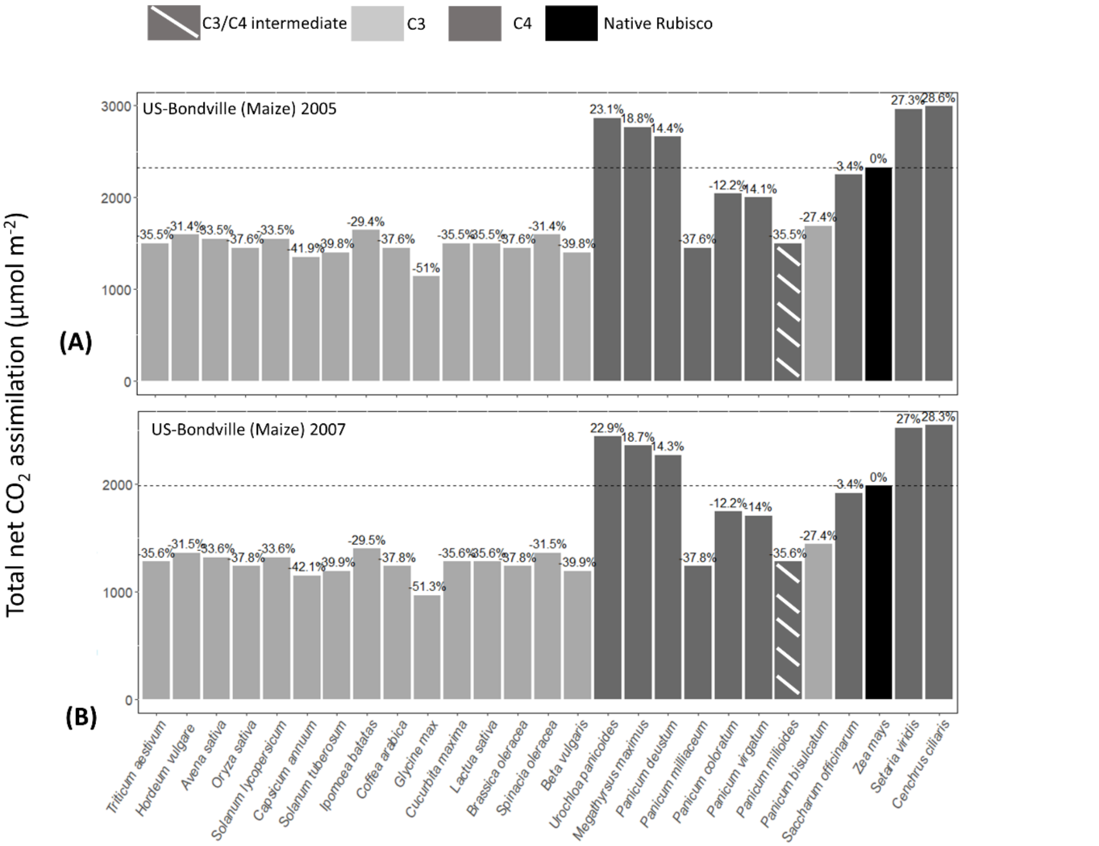

Figure S2. Potential total carbon uptake changes per maize growing season (μmol m^{-2}). Black bars represent maize site simulations with the native Rubisco, and all other bars represent simulations of replacing the native Rubisco with 27 foreign Rubiscos. C_3 plants are shown as dim grey bars, C_4 plants as dark grey bars, and a C_3 / C_4 intermediate patterned. The total carbon uptake of crops with foreign Rubiscos are also shown as a percentage increase or decrease compared to the native Rubisco (dashed lines).

Table S3. Rubisco kinetics measured at 25°C and heat activation (Ha) values used in this study (Sharwood et al., 2016, Hermida-Carrera et al., 2016).

Species	Kcat (s^{-1})	Kc (μM)	Sc/o (mol mol^{-1})	Ha Kcat (KJ mol^{-1})	Ha Kc (KJ mol^{-1})	Ha Sc/o (KJ mol^{-1})
Triticum aestivum	2.2	16	100	41.2	60.4	24.1
Hordeum vulgare	2.4	14.9	99.2	27.9	63.4	21.2
Avena sativa	2.3	18.1	99.9	41.5	63.4	23.6
Oryza sativa	2.1	17.3	93.1	46.4	83.1	24.6
Solanum lycopersicum	2.3	16.6	92.4	34.6	62.1	21.8
Capsicum annuum	1.9	19.8	96	39.2	51.8	24.1
Solanum tuberosum	2	18	95.4	46.2	38.2	24.7
Ipomoea batatas	2.5	21.1	98.5	33.4	42.4	22.8
Coffea arabica	2.1	22.9	98.7	39	71.5	23.4
Glycine max	1.5	16.2	97	55.2	71.1	26.5
Cucurbita maxima	2.2	19.2	98.4	48.7	57	21.1
Lactua sativa	2.2	18.2	94	33.3	55.8	21.2
Brassica oleracea	2.1	19.2	96.2	45.7	55.3	21.8
Spinacia oleracea	2.4	16.9	97	48	69.9	25.2
Beta vulgaris	2	18.6	101	51.2	57	19.8
Urochloa panicoides	5.6	24.1	78.3	57.3	29.9	22.3
Megathyrsus maximus	5.3	27.1	80.3	50.2	22.2	22.2
Panicum deustum	5	28.1	84.8	59.5	28.7	20.8
Panicum milliaceum	2.1	13.1	79.9	71.6	34.9	28.6
Panicum coloratum	3.4	17.3	84.8	58.3	21.3	23.5
Panicum virgatum	3.3	24.5	82.6	58.1	34	23.8
Panicum milioides	2.2	12.1	92.3	68.4	25.4	27.6
Panicum bisulcatum	2.6	12.6	87.7	71.2	31	29.7
Saccharum officinarum	3.9	31.7	82.2	30.2	58.3	25.8
Zea mays	4.1	42	87.3	31	44.5	24.3
Setaris viridis	5.9	25.5	72.7	56.6	21.4	25.3
Cenchrus ciliaris	6	29.2	69.9	54.9	22.1	20.3

Bonan, G. 2019. *Climate Change and Terrestrial Ecosystem Modeling,* Cambridge, Cambridge University Press.

Bonan, G., Drewniak, B., Huang, M., et al. 2013. Technical description of version 4.5 of the Community Land Model (CLM). *NCAR Tech*, -.

Bonan, G. B. 1993. Importance of leaf area index and forest type when estimating photosynthesis in boreal forests. *Remote Sensing of Environment,* 43, 303-314.

Bonan, G. B., Lawrence, P. J., Oleson, K. W., Levis, S., Jung, M., Reichstein, M., Lawrence, D. M. & Swenson, S. C. 2011. Improving canopy processes in the Community Land Model version 4 (CLM4) using global flux fields empirically inferred from FLUXNET data. *Journal of Geophysical Research: Biogeosciences,* 116, G02014.

Burden, R. L. 2016. *Numerical analysis*, Boston, MA : Cengage Learning.

Campbell, G. S. 1974. A simple method for determining unsaturated conductivity from moisture retention data *Soil Science,* 117.

Collatz, G., Ribas-Carbo, M. & Berry, J. 1992. Coupled Photosynthesis-Stomatal Conductance Model for Leaves of C4 Plants. *Functional Plant Biology,* 19**,** 519-538.

Farquhar, G. D., Von Caemmerer, S. & Berry, J. A. 1980. A biochemical model of photosynthetic CO2 assimilation in leaves of C3 species. *Planta,* 149**,** 78-90.

Franks, P. J., Berry, J. A., Lombardozzi, D. L. & Bonan, G. B. 2017. Stomatal Function across Temporal and Spatial Scales: Deep-Time Trends, Land-Atmosphere Coupling and Global Models. *Plant Physiology,* 174**,** 583.

Frouin, R., Lingner, D., Gautier, C., Baker, K. & Smith, R. 1989. A simple analytical formula to compute clear sky total and photosynthetically active solar irradiance at the ocean surface. *Journal of Geophysical Research,* 94**,** 9731-9742.

Goudriaan, J. 1988. The bare bones of leaf-angle distribution in radiation models for canopy photosynthesis and energy exchange. *Agricultural and Forest Meteorology,* 43**,** 155-169.

Hermida-Carrera, C., Kapralov, M. V. & Galmés, J. 2016. Rubisco Catalytic Properties and Temperature Response in Crops. *Plant Physiology,* 171**,** 2549-2561.

Houborg, R., Cescatti, A., Migliavacca, M. & Kustas, W. P. 2013. Satellite retrievals of leaf chlorophyll and photosynthetic capacity for improved modeling of GPP. *Agricultural and Forest Meteorology,* 177**,** 10-23.

Liou, K.-N. 2002. *An introduction to atmospheric radiation*, Elsevier.

Rogers, A., Medlyn, B. E., Dukes, J. S., et al. 2017. A roadmap for improving the representation of photosynthesis in Earth system models. *New Phytologist,* 213**,** 22-42.

Sellers, P. J. 1985. Canopy reflectance, photosynthesis and transpiration. *International Journal of Remote Sensing,* 6**,** 1335-1372.

Sellers, P. J., Berry, J. A., Collatz, G. J., Field, C. B. & Hall, F. G. 1992. Canopy reflectance, photosynthesis, and transpiration. III. A reanalysis using improved leaf models and a new canopy integration scheme. *Remote Sensing of Environment,* 42**,** 187-216.

Sharwood, R. E., Ghannoum, O., Kapralov, M. V., Gunn, L. H. & Whitney, S. M. 2016. Temperature responses of Rubisco from Paniceae grasses provide opportunities for improving C3 photosynthesis. *Nature Plants,* 2**,** 16186.

Sun, Y., Gu, L. & Dickinson, R. E. 2012. A numerical issue in calculating the coupled carbon and water fluxes in a climate model. *Journal of Geophysical Research: Atmospheres,* 117**,** D22103.

Von Caemmerer, S. 2000. *Biochemical Models of Leaf Photosynthesis,* Australia, CSIRO.

Wilson, T. B. & Meyers, T. P. 2007. Determining vegetation indices from solar and photosynthetically active radiation fluxes. *Agricultural and Forest Meteorology,* 144**,** 160-179.

Wright, I. J., Reich, P. B., Westoby, M., et al. 2004. The worldwide leaf economics spectrum. *Nature,* 428**,** 821-827.

Xin, Q., Dai, Y. & Liu, X. 2019. A simple time-stepping scheme to simulate leaf area index, phenology, and gross primary production across deciduous broadleaf forests in the eastern United States. *Biogeosciences,* 16**,** 467-484.
